# Routine screening of Indigenous cancer patients’ unmet support needs: a qualitative study of patient and clinician attitudes

**DOI:** 10.1186/s12939-016-0380-2

**Published:** 2016-06-10

**Authors:** B. Thewes, E. Davis, A. Girgis, P. C. Valery, K. Giam, A. Hocking, J. Jackson, V. Yf He, D. Yip, G. Garvey

**Affiliations:** Menzies School of Health Research, Charles Darwin University, Adelaide Street, PO Box 10639, Brisbane, QLD 4000 Australia; South Western Sydney Clinical School, UNSW, Sydney, Australia; QIMR Berghofer Medical Research Institute, Brisbane, Australia; Alan Walker Cancer Care Centre, Royal Darwin Hospital, Darwin, Australia; Peter MacCallum Cancer Centre, Melbourne, Australia; Southern NSW Local Health District, New South Wales, Australia; ANU Medical School, Australian National University, Canberra, Australia

**Keywords:** Indigenous, Aboriginal, Cancer, Unmet needs, Feasibility

## Abstract

**Background:**

Indigenous Australians have poorer cancer outcomes in terms of incidence mortality and survival compared with non-Indigenous Australians. The factors contributing to this disparity are complex. Identifying and addressing the psychosocial factors and support needs of Indigenous cancer patients may help reduce this disparity. The Supportive Care Needs Assessment Tool for Indigenous People (SCNAT-IP) is a validated 26-item questionnaire developed to assess their unmet supportive care needs. This qualitative study reports on patient and clinician attitudes towards feasibility and acceptability of SCNAT-IP in routine care.

**Methods:**

Forty-four in-depth semi-structured interviews were conducted with 10 clinical staff and 34 Indigenous cancer patients with heterogeneous tumours. Participants were recruited from four geographically diverse Australian cancer clinics. Transcripts were imported into qualitative analysis software (NVivo 10 Software), coded and thematic analysis performed.

**Results:**

Indigenous patients (mean age 54.4 years) found the SCNAT-IP beneficial and easy to understand and they felt valued and heard. Clinical staff reported multiple benefits of using the SCNAT-IP. They particularly appreciated its comprehensive and systematic nature as well as the associated opportunities for early intervention. Some staff described improvements in team communication, while both staff and patients reported that new referrals to support services were directly triggered by completion of the SCNAT-IP. There were also inter-cultural benefits, with a positive and bi-directional exchange of information and cultural knowledge reported when using the SCNAT-IP. Although staff identified some potential barriers to using the SCNAT-IP, including the time required, the response format and comprehension difficulties amongst some participants with low English fluency, these were outweighed by the benefits. Some areas for scaled improvement were also identified by staff.

**Conclusions:**

Staff and patients found the SCNAT-IP to be an acceptable tool and supported universal screening for Indigenous cancer patients. The SCNAT-IP has the potential to help reduce the inequalities in cancer care experienced by Indigenous Australians by identifying and subsequently addressing their unmet support needs. Further research is needed to explore the validity of the SCNAT-IP for Indigenous people from other nations.

## Background

Indigenous people comprise about five percent of the world’s population [1]. Inequalities with regard to Indigenous cancer outcomes have been documented in Australia, New Zealand, Latin America, the Caribbean and the United States [2–6]. In Australia, cancer remains the second leading cause of death among Indigenous people [7]. The patterns of cancer care between Indigenous and non-Indigenous patients differ, with Indigenous patients often receiving less optimal treatment [8–10]. Indigenous Australians’ engagement in cancer care is lower at all stages of the cancer continuum including screening, early presentation at diagnosis, continuity of care, and compliance with treatment [11–14]. This has resulted in significantly poorer cancer outcomes in terms of incidence, mortality and survival.

Aspects of the broader social environment may influence the way individuals, families and communities engage with health care and manage their own health [15]. Understanding Indigenous people’s experience of cancer must take into account the social determinants of health and the cultural context of people’s lives, and these should be reflected in service delivery models and in the delivery of cancer care [16]. Indigenous Australians' collective experience of racism, discrimination, alienation and marginalisation has led to widespread distrust of the health care systems including cancer care and treatment services [15, 17, 18].

It has been widely recognised that there is a lack of culturally appropriate health care services to engage Indigenous Australians [19–22]. Differences in communication, information needs and language have inhibited Indigenous people’s engagement with cancer care [19, 23–25]. This issue is highlighted in a study that was conducted with cancer care professionals, where participants reported doubts about their ability to adequately communicate important information to Indigenous patients [17]. Effective communication between patients and their physicians is essential to ensure optimal patient outcomes [26].

Furthermore, several studies have highlighted cultural differences in the way many Indigenous people perceive cancer (a highly feared disease that equates to death), receive and process information about their cancer diagnosis and treatment, and cope with illness [11, 27–29]. Research into the psychosocial factors and specific supportive care needs of Indigenous people with cancer is imperative.

Improved supportive care for Indigenous cancer patients is an important strategy which may ultimately help narrow current disparities in cancer outcomes.

Many questionnaires have been developed to assess the unmet needs of cancer patients [30]. Some have been developed for specific cancers or settings, but little attention has been given to assessing the appropriateness of existing tools for minorities or developing tools that assess the culture-specific needs of different ethnic groups [30]. In Australia, Indigenous cancer patients share many supportive care needs with non-Indigenous cancer patients, but they have additional culture-specific needs that are not addressed by existing measures [31]. To address this gap, a Supportive Cancer Needs Assessment Tool for Indigenous People (SCNAT-IP) has been developed [31, 32].

The broader clinical implementation feasibility study has been reported elsewhere [33]. While integrating qualitative and quantitative data can complement and deepen the interpretation of findings, we justify presentating the qualitative data separately on the basis of: (a) the paucity of research involving Indigenous cancer patients and in particular studies which explore unmet support needs; (b) the richness of the qualitative data; and (c) being able to illustrate the range and depth of participants needs and concerns in their own words rather than by a summary of themes. This study describes patient and staff attitudes towards the acceptability and feasibility of the SCNAT-IP in routine care. Additionally, this study aimed to identify refinements needed to prepare the SCNAT-IP for use in clinical settings.

## Methods

### Ethical considerations, Study sites and participant eligibility criteria

Ethical clearance was obtained from the following Human Research Ethics Committees: Northern Territory (NT) Department of Health & Families and the Menzies School of Health Research (HREC13/1994); Peter MacCallum Cancer Centre (HREC/13/45); Greater Western Human Research Ethics Committee (HRECI131GWAHSI39) and the Aboriginal Health and Medical Research Council of New South Wales (NSW; HREC/946/12). The content of this manuscript was subject to approval by the Aboriginal Health and Medical Research Council of NSW in accordance with the standard conditions of their ethical approval.

Participants were recruited from one of four participating sites: a tertiary cancer centre in the NT servicing outer regional and remote areas; a large metropolitan tertiary cancer centre in Victoria; and two regional cancer clinics in NSW. These four services represented a diversity of geographical locations, service models and Indigenous communities. All participating sites had a relatively high proportion of Indigenous patients, and/or dedicated Indigenous hospital liaison staff or an interest in improving service delivery for Indigenous patients.

Eligible patients were over 18 years of age, of Australian Indigenous background, were diagnosed five years ago or less with malignant disease, and were either receiving or about to receive treatment (including surgery, chemotherapy, radiotherapy, stem cell transplant, endocrine therapy or immunotherapy) or were in follow-up care. Recruitment was conducted over a three to five month period (November 2013-March 2014). Interviews were verbally administered, and health care interpreters were available upon request by staff at the NT site where many Indigenous people speak a language other than English. Those unable to give informed consent due to cognitive and/or physical impairments were excluded. Eligible cancer nurses, oncology social workers, Indigenous health workers (IHWs) or other allied health professionals were identified by their manager as providing supportive care to cancer patients and invited to participate in the training for this study.

### Procedure

After written informed consent was obtained, all health professionals involved in this study completed a specifically developed three hour training session, which included background information, clinical use of the SCNAT-IP and the research protocol. Following training, all staff at participating sites were asked to identify and refer all eligible Indigenous cancer patients to trained clinical staff for needs assessment. Trained clinical staff (oncology social workers, nurses and clinical trials coordinators) introduced the study to patients, gained written informed consent and conducted needs assessment at routine patient clinic visits. Where moderate to high levels of needs were identified (i.e. scores of four or five on any SCNAT-IP item), these were discussed with patients and they were offered assistance in accordance with usual care.

Following needs assessment, patients, along with a trained clinical staff member, completed quantitative questionnaire items exploring the acceptability and feasibility of the SCNAT-IP; these results are reported elsewhere [33]. Immediately following this, a trained IHW (or a trained clinical staff member if the IHW was unavailable), conducted an audiotaped face-to-face or telephone interview which aimed to gather qualitative information about patient attitudes towards unmet needs screening. Telephone interviews were offered for pragmatic reasons (e.g. when patients' transport was scheduled immediately after the completion of their appointment).

At the conclusion of the data collection period, trained clinical staff completed a brief telephone interview with a member of the research team (BT). Recruitment continued until no new themes emerged from the data. Patients' clinical details were collected by clinical staff from medical records.

#### Measures

### Patient clinical and demographic characteristics

Socio-demographic variables were collected by interview. Clinical details including cancer type, disease status, treatment phase, and cancer treatments were collected from medical records.

### Supportive care needs assessment tool for indigenous people (SCNAT-IP)

The SCNAT-IP is a 27-item (including one open-ended question) verbally-administered unmet needs measure. It consists of four domains: physical and psychological needs (11 items); hospital care needs (four items); information and communication needs (six items) and practical and cultural needs (five items). Items included but were not limited to the following: ‘feeling tired, feeling down or sad’ and ‘keeping you strong in your spirit’ (physical and psychological needs domain); ‘having hospital staff show sensitivity to and respecting your feelings and emotional needs’; and ‘being treated like a person not just another case or number’ (hospital care needs domain); ‘being shown or given information (e.g., written, diagrams) about how to manage your illness and side effects at home’ (information and communication needs domain); and ‘finding a place to stay while receiving treatment and money worries’ (practical and cultural needs domain) [32].

For each item respondents report a yes/no response. If a need was present in the past month they were asked to choose the degree to which they required help on a scale ranging from ‘satisfied because my needs were met’ (scored as one) to ‘a lot more help needed’ (scored as five). To assist with comprehension, participants were given a hard copy of response categories and the interviewer recorded participants' responses [31]. The SCNAT-IP accommodates the language, customs and culture-specific needs of Indigenous Australians and excludes items which are culturally-inappropriate in the screening context (e.g. sexual needs). The tool also includes culture-specific items such as having an Indigenous person to talk to and support you, someone who understands your culture [31]. It takes approximately 15-20 min to complete and has been demonstrated to have good construct and face validity as well as internal consistency in a large sample of Indigenous cancer patients [31, 32].

### Patient acceptability interview

A brief semi-structured face-to-face interview was used to qualitatively explore the participants’ attitudes with regards to needs assessment. Interview questions explored perceived benefits of needs assessment, acceptability, appropriateness of content and timing, and attitudes towards universal screening for Indigenous patients.

### Health professional characteristics

Items were developed to assess demographic characteristics, role, education, employment location, years of experience and prior supportive care needs assessment training.

### Health professional acceptability and feasibility telephone interview

Six semi-structured interview questions assessed perceived benefits, barriers to use, impact on workload, impact on team communication/referral pathways, and recommendations for changes to the SCNAT-IP.

#### Data analysis

Demographic data were analysed using descriptive statistics. Interview audio recordings were transcribed verbatim. Data analysis was performed using QSR International's NVivo 10 Software [34]. Thematic analysis was performed in six phases including: familiarisation with data; generation of initial codes; searching for themes among codes; reviewing themes; defining and naming themes; and synthesising the final results [35]. The generation of coding was conducted by one member of the research team (BT), and verified by a second member of the research team (GG) who double-coded 10 % of transcripts to ensure inter-coder agreement. Where discrepancies arose in coding or definition of themes, these were resolved through discussion until consensus was reached.

## Results

### Response rate and patient characteristics

**Fig. 1:**
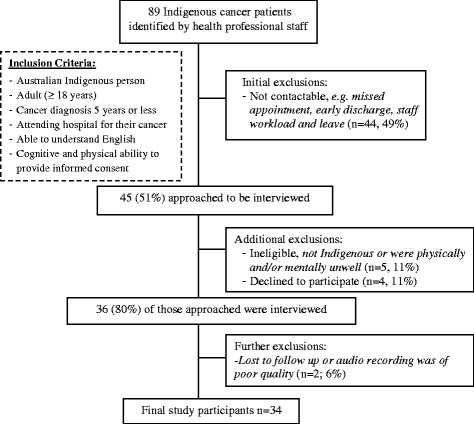
Flow of patient participant recruitment

Of the 89 potentially eligible participants, 45 (51 %) were invited to participate in the study. Thirty-six (80 %) of these agreed to be interviewed and two participants were further excluded leaving a final study sample of 34 (76 %) (Fig. 1).

Participants were aged 34-76 years (mean=54.4, SD=11.0) and the majority were female (68 %), partnered (53 %), living outside metropolitan cities (89 %) and had not completed high-school (60 %). Breast cancer was the most common cancer type (35 %); 31 % had local disease and 47 % were receiving active treatment (Table 1).

**Table 1:** Demographic and clinical characteristics of the patient sample (*n* = 34)

	*n*	%^a^
*Sex*		
Male	11	32
Female	23	68
*Remoteness of residence* ^*b*^		
Major city	4	12
Regional	24	71
Remote	6	18
*Marital status*		
Single	10	29
Married/De Facto	18	53
Widowed/Separated/divorced	6	18
*Main language spoken at home*		
English	19	56
Indigenous language	15	44
*Education level*		
Junior High School or below	21	62
Senior High School	5	15
Diploma/Trade/University degree	8	12
*Cancer Type*		
Breast	12	35
Colorectal	6	18
Head & neck	6	18
Lung	3	9
Gynaecological	2	6
NHL^c^	1	3
Haematological	1	3
Other	3	9
*Cancer Stage*		
Local disease	11	32
Regional spread	5	15
Distal metastases	12	35
Not applicable	3	9
Not known	3	9
*Treatment status*		
Newly diagnosed	4	12
Receiving treatment^d^	16	47
Follow-up care	14	41
*Treatment type*		
* Surgery*		
Completed	22	65
Planned	3	9
No/Not applicable	9	26
*Chemotherapy*		
Yes	22	65
No	12	35
*Radiotherapy*		
Yes	21	62
No	13	38
*Other cancer treatments* ^*f*^		
Yes	13	38
No	21	62
*Comorbidities*		
Diabetes	8	24
Cardiovascular	9	27
Respiratory	9	27
Other^e^	15	44

Notes: ^a^May not sum to 100 % due to rounding; ^b^classified according to Accessibility/Remoteness Criteria of Australia (ARIA) for further information see AIHW [15]. ^c^Non-Hodgkins Lymphoma; ^d^includes all active hospital-based cancer treatments; ^e^includes: Substance Abuse, Psychiatric, Renal disease, Neurological and Gastrointestinal disorders; ^f^Includes: hormonal therapy, brachytherapy, microwave ablation

### Health professional participants and response rate

The SCNAT-IP study training session included 22 health professionals with an average age of 42 years (range 25-62, SD=11.4) and a diverse set of professional backgrounds including social workers (*n* = 8), nurse care coordinators (*n* = 4), radiation therapist (*n* = 1), clinical trial coordinator (*n* = 1), IHWs (*n* = 5) and service managers (*n* = 3). Ten of the 22 session participants conducted needs assessments using the SCNAT-IP. All were non-Indigenous females; six were oncology social workers; nine had post-graduate qualifications; and six had no previous needs assessment training. Half had six years or more experience in cancer care and worked in regional locations. The other 12 health professionals conducted follow-up interviews only or supported the study as service managers.

#### Patient interviews

The results are reported for the combined group because participant views were common across genders, age groups, cancer types, cancer centres and mode of interview. Insufficient patients were interviewed by phone to formally explore whether the mode of interview influenced the results obtained.

### Feasibility and acceptability

The majority of participants liked being asked about their needs, did not mind taking the time to answer questions and generally found the process very helpful.

Some said they liked the process of needs assessment because it made them feel heard and it linked them to services. As one participant who lived in a regional area said:

*“This is just a god send to me because now I know I’m going to get some help because it’s always been a real battle for me.”*

(Female, 50 years)

Another female participant reported feeling “*empowered*” by the process of needs assessment. Several participants appreciated that health professionals were interested in finding out about their needs and that they showed an interest particularly in the needs of Indigenous cancer patients.

*“It’s made me feel good that someone’s showing interest, you know. That there's people out there who are trying to improve things, not only for cancer patients but for Indigenous people.”*

(Male, 45 years)

Only one participant reported negative feelings about the SCNAT-IP, saying she felt “*uptight*” being asked about her unmet needs but later clarified “*cause [I] told her [health professional] a few things that she didn’t know*.” (Female, 55 years) Another participant did not find the process of needs assessment helpful because they had visited the centre many times in the last seven years, commenting “*we’re pretty well used to it*”, but further remarked “*these sort of questions would have been more helpful in the beginning*” (Male, 75 years).

### Format and language

Most participants found the tool very easy to understand and all liked the format of being asked the questions by health professional staff. No participants directly reported problems of comprehension.

### Ideal timing and frequency of SCNAT-IP screening

This sample included patients at all stages of the treatment continuum, from diagnosis to follow-up care. Most participants reported being satisfied with the timing of the questionnaire, although several said it would have been more useful if completed earlier in their cancer journey.

*“To be perfectly honest it would have been helpful while I was having treatment done. It’s a little bit late because my last appointment was today.”*

(Male, 35 years)

Another participant at the beginning of her treatment for breast cancer said the questionnaire should be completed closer to the time of diagnosis, whilst another remarked these questions should be asked early so they have “*some information about it, [and are] not left in the dark.*”

(Female, 41 years)

However, a newly-diagnosed male participant suggested that SCNAT-IP assessment should not occur too soon after diagnosis because some subjects were “*a bit touchy*” because they were still coming to terms with the shock of their very recent cancer diagnosis.

Although most patients favoured early administration, there were diverse views about the ideal frequency, ranging from “*once a week*” or “*every time I go in* [to the cancer centre]” to “*every six months*” or “*every* [cancer treatment] *stage*”. The majority of participants believed the SCNAT-IP assessment should be repeated throughout treatment.

### Perceived benefits of services offered following screening

All participants with moderate to high needs (*n* = 21, 58 %) were offered services to help address unmet needs and many reported experiencing benefits.

*“Yes, she* [social worker] *gave me some information because of some of my answers. I don’t think I would have got that information otherwise.”*

(Female, 51 years)

*“I have now been referred to psychology and what I said is, “I wish this had have happened two years ago.”*

(Female, 55 years)

There was also a real sense of appreciation and trust felt by participants of the staff asking these questions, as one participant described, “*it was good to be asked…[even though]… I do have a lot of help from my family”* (Female, 49 years) and another commented “…*a lot of that which build up in you, you like to talk to somebody about it, but it’s hard, hard for people just to listen*” (Female, 60 years). Another participant from a more rural location commented “*I’m not going back there [home]…apprehensive and worried and scared to what I usually have been….[social worker] is going to organise all the things. So when I go back it will all sort of follow; so there will be no stress. All I would need to worry about is just getting well.”*

(Female, 50 years)

### Attitudes to universal screening

Many patients supported the idea of universal screening for Indigenous cancer patients noting that it would educate staff about Indigenous patient needs.

*“A lot of them [staff] don’t understand there is problem like family problem, money problems.”*

(Female, 47 years)

Others said it helped educate patients about what help is available to them and what to expect during treatment. Some participants believed needs screening should be optional to promote control and self-determination.

*“Give ‘em the choice, you know, ‘cause a lot of ‘em feel like they’re already being controlled by other people in their lives as it is and they don’t need, ‘specially in a place like this, they don’t need to get a view of it like 'okay here’s another government organisation that’s gonna be telling me what to do'".*

(Male, 45 years)

Some participants without current unmet needs believed needs assessment was more worthwhile for others for altruistic reasons.

*“I’m pretty articulate and pretty aware of what the processes are within hospitals and so forth, but for somebody else you know, I think it’s something that needs to be done.”*

(Male, 61 years)

Whilst being generally satisfied with the SCNAT-IP and willing to complete it, some participants believed that for other Indigenous people needs screening is important because of cultural factors such as being "*shy*" and “a reluctance to talk about problems or articulate their needs”.

Despite the SCNAT-IP being specific to Indigenous people, several participants said that SCNAT-IP would be relevant for all cancer patients irrespective of race. Two participants interviewed did not support universal screening for Indigenous people with cancer, one because she believed some questions were “*too personal”* and a second said he was “*not sure*” why.

“*They might think that you’re violating that little part of their life that they can hold tight.*”

(Female, 60 years)

### Staff qualitative data

#### Perceived benefits of screening

Many staff said there were multiple benefits of the SCNAT-IP, including its comprehensiveness, its systematic approach to needs assessment and the opportunities for early intervention which other less formal assessments methods did not offer. Using the SCNAT-IP was also reported to help staff increase their awareness and identification of Indigenous patients in their clinic.

*“It picks up things that I think would never have come up until we were at a real crisis point”.*

(Oncology social worker)

Several staff said it helped to build rapport, made patients feel “*heard”*, and helped clarify patient expectations of services. Some staff reported they liked using an Indigenous-specific questionnaire because it fostered positive and collaborative relationships with Indigenous patients.

*“The feedback I’ve got from patients was they’ve certainly felt like they were heard which I think is something Aboriginal patients here have found difficult in the past.”*

(Oncology social worker)

*“I think it allows people then to have a connection with the social work team here as well. I’ve found that people that may otherwise not have had any contact with the social workers here have been able to make contact in a way that has been positive.”*

(Oncology social worker)

Staff also identified that using the SCNAT-IP helped educate staff members less familiar with working with Indigenous clients about culturally-specific needs and services.

*“I think it’s also a good opportunity for staff who may not be as experienced or, you know, trained for working with people of Indigenous background to have this sort of specific tools to use. It can make some people a little bit more comfortable being able to ask some of these questions otherwise they may not know where to start or the right language to use.”*

(Oncology social worker)

The verbal format of the tool was seen as a distinct advantage over other written scales.

### Barriers to use of the SCNAT-IP in routine care

The most commonly identified barriers to using the tool were the time needed to arrange an appointment and to complete the interview at the patients' pace. SCNAT-IP interviews (including follow-up discussion of needs) lasted between 6 and 45 min with an average duration of 23 min.

*“As much as we want to sit and have a really good yarn with people, having so many questions in it [the questionnaire]... it probably encourages them to yarn a lot more…I think there’s the potential to be a lot of breaks in between the questions for yarning and you need to allow a bit more time”.*

(Cancer care coordinator)

Logistical problems were especially common for rural or remote patients because many attend clinics infrequently and have long travel times to consider, and this impacted on their ability to allow sufficient time to conduct the assessment.

Difficulties with comprehension of the tool were only reported at one site where many patients spoke an Indigenous language as their primary language. Despite health care interpreters being available upon patient or staff request at this site, many patients preferred to conduct interviews in English and relied on accompanying family members and/or staff to assist with translation.

Some staff reported that some patients had difficulty distinguishing between lower levels of need (e.g. ‘a little more’ versus ‘some more’ help), hence requiring further clarification by staff. Rapport building and allowing plenty of time to complete the interview were reported as having helped to reduce comprehension difficulties. Several staff also reported that some questions were not relevant to patients because of the time frame (i.e. ‘in the past month’) because of different patients’ stages in the treatment trajectory (e.g. hospital items for newly-diagnosed patients).

Whilst one staff member identified a cultural tendency for Indigenous people to be private or reluctant to disclose personal information as a potential barrier to using the SCNAT-IP, another staff member saw the structured and direct nature of the tool as a benefit in this respect.

*"Often in my experience Indigenous people are quite private and they’ll say things are fine or they don’t need any help but it’s good actually having those questions as prompts."*

(Cancer care coordinator)

### Impact on workload

The majority of staff said that completing the questionnaire took additional time but there were concomitant time savings because they had a better understanding of patient needs and could therefore provide early intervention.

*“I think it definitely makes up for it later on because you can activate services a bit earlier.”*

(Oncology social worker)

Some staff reported it reduced their workload. Others said it increased workload but this was sometimes appraised in a positive way.

*“I mean it’s… it’s increased my workload but that’s ok, I see that as fine. It’s increased my workload but that’s good practice. It’s part of doing a thorough assessment with people with cancer and being aware of unmet needs.”*

(Oncology social worker)

Experience using screening tools was reported to reduce the time required. One staff member believed the impact on workload would be less if the questionnaire were conducted at the time of initial service entry when other questionnaire information is routinely gathered.

### Team communication

Several benefits to team communication were reported by staff including improved communication with IHWs, and nursing and medical staff.

*“It’s probably given me the confidence to liaise more with our Aboriginal liaison officers which I haven’t done in the past very much.”*

(Cancer care coordinator)

One staff member said that using the SCNAT-IP identified gaps in patients’ understanding of their diagnosis or treatment. This staff member reported that the SCNAT-IP:

*“helps the doctors here understand how they need to change the way they explain things to their Indigenous patients”.*

(Oncology social worker)

Making SCNAT-IP results available to nursing and medical staff in patient files was reported to facilitate better communication about patients’ psychosocial needs. One staff member believed there were greater opportunities for the SCNAT-IP to have a positive impact on team communication if it were used early in the treatment trajectory.

### Impact on referral pathways

Many staff reported that using the SCNAT-IP triggered new supportive care referrals. One clinician from a metropolitan hospital, who interviewed a number of patients from regional areas, observed that completing the SCNAT-IP increased her awareness of her need to better understand referral networks in regional areas. However, staff based in regional clinics observed little or no impact on referral pathways. This was attributed to the relatively small numbers of Indigenous patients who were assessed in those clinics and the fact that most of their patients were not newly-diagnosed and were already linked to local support services.

### Suggested changes

Several staff members suggested minor changes to the structure, wording or format of the SCNAT-IP tool including reducing the number of items, further simplifying the wording and modifying the response time frame (i.e. ‘*in the past month’*) to make it more applicable across the whole treatment continuum. It was also suggested to group items by domain and use domain headings to help clarify item content, as well as revise the preamble to clarify the purpose and response format (i.e. use of emoticons), and highlight the fact that some items were not relevant to all patients. More advice for staff members about the time required to complete the instrument and follow-up discussion about needs was also recommended. Contradictory views about who should complete the assessment were noted, with one staff member suggesting wider use, for example, by treating doctors or ward nursing staff, and another expressing reservations as to whether the medical or ward staff would be the best team members to assess and respond to unmet needs.

## Discussion

Overall patient acceptability of the SCNAT-IP was high, with the majority of participants finding the tool easy to understand. They liked the format and appreciated being asked by health professionals about their supportive care needs. These results are consistent with the results of a quantitative analysis of acceptability and feasibility in this sample [33]. They also align with the results of patient and key informant interviews conducted during the development of the SCNAT-IP [31] and with past research that demonstrated the acceptability of the unmet needs assessment screening amongst the general Australian cancer population [30]. Both patients and staff in this study believed SCNAT-IP assessment would be most useful early on in the treatment trajectory, with reassessment throughout treatment tailored to individual need.

Indigenous patients' cultural disconnection within the healthcare system is well documented [11, 24, 36] and the favourable results regarding the patient acceptability of the SCNAT-IP may simply have been due to health professionals taking more time to sit and talk with patients. However, our results indicate that it was more than this. Patients liked being asked about their supportive care needs in a structured manner and they reported the process was very helpful because this had not occurred prior to the implementation of the SCNAT-IP. The SCNAT-IP also provided health professionals with the means to have these discussions in a culturally appropriate format and they further commented that the tool picks up things they would not have identified if they were simply taking the time to sit and talk with the patient.

Basic infrastructure and logistical issues, such as lack of transport and suitable accommodation, are also reported to impede Indigenous Australians’ access to cancer care and treatment services [11, 15, 23, 37]. The use of the SCNAT-IP is intended to assist health professionals to identify and subsequently address these important practical and logistical issues to improve access and uptake of cancer care.

Whilst this study provides additional evidence for the feasibility and acceptability of the SCNAT-IP, some staff identified comprehension difficulties amongst patients with low English literacy as a potential barrier. However, patients who spoke Indigenous languages at home did not report any comprehension difficulties [33]. Reasons for this discrepancy are not clear. Although in most parts of Australia less than 10 % of the Indigenous population speak an Indigenous language at home (in the NT this figure rises to over 60 %) [38], language and literacy are commonly reported to be barriers to accessing health care and support services [24, 28]. These barriers may be due to Indigenous patients’ lack of cancer knowledge and confidence in communicating with health professionals or navigating the health system, as well as cancer care professionals not tailoring their language to ensure that Indigenous patients fully comprehend their diagnoses, prognoses and treatment options [16, 39]. Given the verbal nature of the SCNAT-IP and the fact that most clinicians are non-Indigenous, health care interpreters are a preferred delivery method. Despite trained health care interpreters being available in this study they were under-utilised. Using family members as interpreters is not recommended practice when health communication is of a complex or sensitive nature because it can lead to serious misunderstandings [40]. Our study did not formally assess participants’ need for an interpreter or specifically explore the relationship between language spoken at home and time required to complete the instrument. Future studies should consider using more formal methods of assessing need for an interpreter and/or consider mandating use of trained health care interpreters for participants who primarily use Indigenous languages.

This trial included sites servicing Indigenous people living in metropolitan and regional areas, as well as those from remote communities. Our study did not assess geographical variations because participants residing in remote and metropolitan areas were under-represented in the sample. However, participants were recruited from four diverse cancer services which represented a range of geographical locations, service models and Indigenous communities. Our aggregated study data suggests that the SCNAT-IP is acceptable and feasible for use in a variety of service settings. Given the small number of participants from remote and very remote communities in this study, this finding should be interpreted with caution and further research involving those from remote and very remote communities is required. A number of Indigenous people were excluded from this study due to individual and system related issues such as patients not turning up for appointments, competing priorities, incorrect Indigenous status recorded on patient medical records, difficulties scheduling appointments and travel issues. These issues may represent unmet needs beyond the scope of this study and the SCNAT-IP, and could also be important in improving Indigenous patients’ health outcomes. These individual and systemic issues affect Indigenous health more generally, but none-the-less impact on the delivery of cancer care and potentially the health outcomes of Indigenous Australians.

Although conducted in Australia and involving Indigenous Australians exclusively, this is the first qualitative study to explore feasibility and acceptability of an Indigenous-specific psychosocial screening tool. Further research is needed to explore the validity and possible adaption of the SCNAT-IP for other Indigenous groups.

An interesting and somewhat unexpected finding of this study was the inter-cultural benefits of the SCNAT-IP, with a positive and bi-directional exchange of information and cultural knowledge reported, potentially leading to improved inter-cultural collaboration and relationships that come from using a culture-specific tool like the SCNAT-IP. Whilst not the focus of this study, future studies should consider expanding study outcomes to include broader cultural benefits in addition to the more traditional service delivery outcomes and patient self-report measures which have formed the focus of many previous screening trials.

In considering the findings of this study some limitations should be acknowledged. First, although the study aimed to recruit all eligible Indigenous cancer patients seen during the study period, more than half of all potentially eligible Indigenous cancer patients were not invited to participate in this study for the reasons outlined above. Second, a small number of patients identified in medical records as being Indigenous did not self-identify as Indigenous when invited to the study. Third, inaccuracy of medical records to identify cancer patients of Aboriginal and Torres Strait Islander background may have influenced the results. Fourth, although the majority of participants had high levels of English fluency and did not require interpreters, it is likely that the routine use of interpreters amongst those who spoke Indigenous language(s) as their primary language would have increased the range and richness of responses for those patients. Fifth, the early onset of the wet season in the NT impeded patient transport from remote communities to the clinic, which reduced overall patient numbers during this trial. As a result we may have missed the views of those most marginalised and hardest to reach due to distance and other barriers not mentioned (cost, competing priorities). Finally, although the study intentionally included participants from a range of geographic areas, we acknowledge that further research is needed with larger numbers of participants from remote and metropolitan areas to ensure the generalisability of the findings. Despite these potential biases which may impact on validity, this study had a high recruitment rate and a relatively large sample size for qualitative studies.

This study confirmed the SCNAT-IP is applicable to both the research and clinical cancer care settings. For clinical purposes, our findings suggest that the SCNAT-IP should be used at a point early in the treatment trajectory with reassessment at subsequent time points dependent on individual patient need. Overall, the SCNAT-IP will greatly assist cancer care providers to identify Indigenous cancer patients’ unmet needs and priorities for action. Further, it will also assist cancer care providers to assess the adequacy of their services in addressing patients’ needs and identify areas for service improvement. Additional benefits include enhancing patient-provider communication and improving patients’ experience and satisfaction with cancer care. Ultimately the goal is to bring about positive changes to patient management and improve cancer outcomes for Indigenous Australians.

## Conclusions

This study provides further evidence for the feasibility and acceptability of the SCNAT-IP and its potential to help reduce the inequalities in cancer care experienced by Indigenous Australians. The results have led to minor modifications to the SCNAT-IP to enhance its clarity and suitability for use in a variety of settings. Strategies to promote and disseminate the SCNAT-IP are currently underway. Future studies should consider using more formal methods of assessing need for an interpreter and/or consider mandating use of trained health care interpreters for participants who primarily use Indigenous languages.

## Abbreviations

SCNAT-IP: the supportive care needs assessment tool for Indigenous people
